# A giant jejunal gastrointestinal stromal tumor misconceived as pancreatic cystic neoplasm: A case report

**DOI:** 10.1016/j.ijscr.2019.06.023

**Published:** 2019-06-17

**Authors:** Kitti Wongta, Vorapatu Tangsirapat, Vichack Chakrapan Na Ayudhya, Papot Charutragulchai, Singha Sripreechapattana, Kobkool Chakrapan Na Ayudhya, Rapol Poolsavatkitikool, Paiboon Sookpotarom, Paisarn Vejchapipat

**Affiliations:** a Department of Surgery, Panyananthaphikkhu Chonprathan Medical Center, Srinakharinwirot University, Nonthaburi, 11120, Thailand; b Department of Surgery, Faculty of Medicine, Chulalongkorn University, Bangkok, 10330, Thailand

**Keywords:** GISTs, Pancreatic tumor, Jejunum, Case report

## Abstract

**Introduction:**

Gastrointestinal stromal tumors (GISTs), although not common in gastrointestinal (GI) tumors, constitute the most frequent mesenchymal tumors of the GI tract. This report describes a patient with a large sporadic GIST at proximal jejunum that mimicked the pancreatic cystic neoplasm.

**Case presentation:**

We report a 59-year-old female patient with unexplained weight loss and palpable left upper quadrant abdominal mass for 6 months. Computed tomography (CT) scan demonstrated a heterogeneously mass measuring 10 cm in a maximal diameter at pancreatic body and tail. Laparotomy was done, and the tumor was found at proximal jejunum closed to the ligament of Treitz, so en bloc resection of the tumor was done. The pathological and immunohistochemical study confirmed GISTs.

**Discussion:**

Although most GISTs are originated from the intestine, they frequently arise from the stomach, the duodenum and rarely from the jejunum. And jejunal GISTs are usually asymptomatic. The misdiagnosis as mucinous cystadenoma of this case might be due to the proximity of the tumor to the body and tail of pancreas, and compressing the adjacent organ due to its large size.

**Conclusion:**

GISTs are most frequently small and arising from the stomach. GISTs are rarely present as a large tumor of the jejunum, and particularly mimicked a pancreatic tumor.

## Highlights

• Large GIST in proximal jejunum mimick the mucinous cystic neoplasm of pancreas by preoperative Computed Tomography (CT) scan.
• GISTs are rarely occurred in jejunum.
• The misdiagnosis of this case might be due to the proximity of the tumor to the body and tail of pancreas, and compressing the adjacent organ due to its large size.

## 1 Introduction

Gastrointestinal stromal tumors (GISTs) are considered as rare neoplasms in gastrointestinal (GI) tumors; however, they are the most common mesenchymal tumors of the GI tract [1]. GISTs, most frequently arising from stomach, rarely occurred at the small intestine, particularly at jejunum [2]. They are usually small and develop ulceration that proceeds to GI bleeding. Interestingly, our patient had an unusual presentation with a very large tumor arose from the jejunum. Not only there was an absence of GI symptoms, but the tumor initially was misconceived as a cystic lesion of the pancreas.

This work is compliant with the SCARE checklist, and also, has been reported in line with the SCARE criteria [3].

## 2 Case presentation

**Fig. 1:**
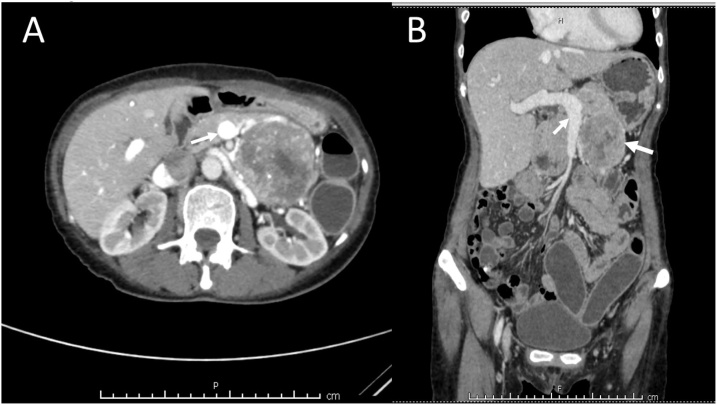
Axial (A) and coronal (B) view of contrasted abdominal CT showed heterogeneously enhanced solid-cystic tumor (larger arrow) abutted onto a great vessel (smaller arrow).

A 59-year-old female patient has encountered a problem of unexplained weight loss over 10 kg within 6 months. Except for a nonspecific abdominal pain, there were no other GI symptoms. Her vital signs were within normal limit. On examination, she was pale. It was astounding for the patient that there was a palpable fist-sized mass at left upper quadrant during the physical examination. The routine laboratory tests revealed hemoglobin of 9 g/dL, and other blood tests were unremarkable. Computed tomography (CT) scan demonstrated a heterogeneously enhancing solid-cystic mass measuring 10 cm in maximal diameter at the pancreatic body and tail (Fig. 1). The provisional diagnosis of pancreatic mucinous cystadenoma was made and the patient was scheduled for distal pancreatectomy, lymphadenectomy, and splenectomy.

**Fig. 2:**
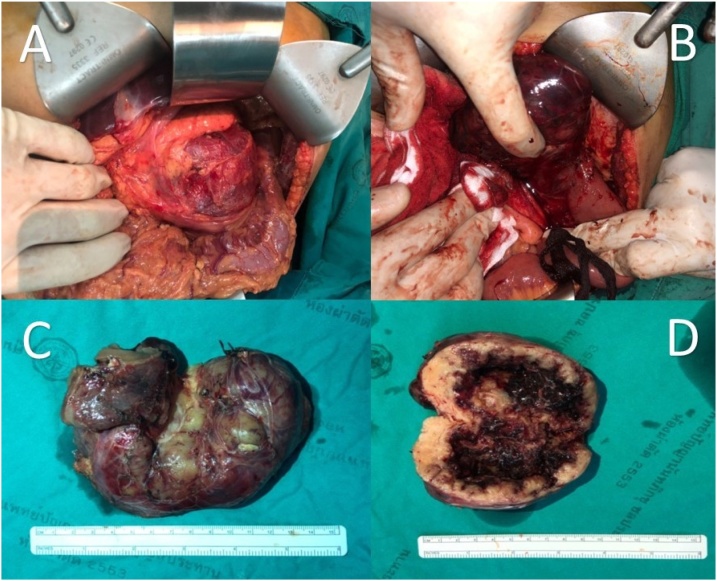
The jejunal GIST was bluntly dissected from pancreas (A and B). The tumor originating from proximal jejunum was soft, round shaped, and tan-grey colored (C). There was a large central necrosis and cavity on cut surface (D).

**Fig. 3:**
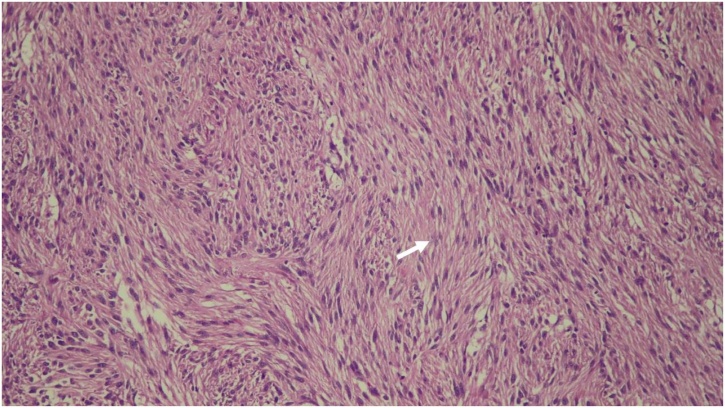
Serial sections showed an intramural mass consisting of bundle of spindle cells with interlacing bundle formation. There were mild nuclear atypia and scant mitosis(Arrow).

At the theatre, the exploration through a Chevron incision revealed that the tumor originating from the proximal jejunum just distal to the ligament of Treitz, in lieu of a tumor of the pancreas (Fig. 2). The tumor that was abutting the body and tail of pancreas, with meticulous handling, was dissected and removed. There was no spillage or rupture of the capsule while removing the tumor. End-to-end jejunojejunostomy completed the operation. There was an absence of adjacent mesenteric lymphadenopathy. Gross specimen, measuring 9 × 8 × 6.5 cm, was a soft, round-shaped, tan-grey colored mass with an irregular surface. There was large central necrosis and cavity. With a sequential serial section on histologic examination, the tumor was contained in the small intestinal segment in which the intramural mass consisted of interlacing bundle of spindle cells and interlacing bundle formation (Fig. 2). Nuclear atypia was presented. However, nuclear mitosis was scarcely found (0–1 /high-power field) (Fig. 3). The tumor margins were free from tumor cells. In the immunohistochemical study, the tumor cells were positive for tyrosine-kinase protein (CD117) and transmembrane protein (DOG1) which was consistent with GISTs. Post-operatively, there was no complication. The patient uneventfully recovered and discharged on postoperative day 8. Due to the large-sized tumor which entails an increased risk of tumor recurrence, adjuvant therapy was initiated with imatinib mesylate.

The patient was symptom-free during follow up at one month, three months, and six months after surgery. The CT scan was performed one year after the operation and no recurrence was detected.

## 3 Discussion

GISTs constitute the most common mesenchymal tumor of the GI tract, although their incidence is very low compared with those of all other tumors arising from the GI tract, they are most frequently found at the stomach. Though the tumors originated from the intestine, they usually arise from the duodenum and rarely from the jejunum [4]. Generally, patients with GISTs present with a symptom of GI bleeding as a primary complaint. In contrast, jejunal GISTs are usually asymptomatic. Likewise, our case presented only with nonspecific abdominal pain and unexplained weight loss. Perforation of the jejunal GISTs has been reported, in which all of them presented with acute abdominal pain and generalized peritonitis [1,[5], [6], [7]]. Fortunately, in spite of a large tumor, the patient has not encountered an unfortunate event.

CT scan typically demonstrates GISTs as a heterogenous occupied lesion with high vascularity [8]. The large tumor may have a central necrotic area and intratumoral hemorrhage. However, the definite diagnosis usually derived from a concluding pathologic report. Interestingly, in the report of this case, the CT scan revealed an enhancing solid-cystic tumor obviously originated from the pancreatic body and its tail. In addition, there was no apparent plane of separation between the two structures. Consequently, they were construed as a tumor of the pancreas rather than others. The reasonable explanation for the provisional diagnosis of mucinous cystadenoma might be its proximity to the body and tail of pancreas and its large size causing the tumor to compress the adjacent organ.

There are several antibodies that express in GISTs [9,10]. However, it seems that CD117, a marker of intestinal Cajal cells, and DOG1, primarily discovered on GISTs, are the antibodies of choice in the diagnosis of GISTs [11]. Following the routine pathologic report, the patient’s specimens were positive for CD117 and DOG1 confirming the diagnosis of GISTs. Presently, surgical tumor removal is the only potentially curative therapy for patients with primarily resectable GISTs [12]. A small-sized tumor with low mitotic activity indicates low-risk tumor, predicting more favorable prognosis [12]. However, the presence of a large-sized tumor categorized this patient into a high-risk group. Since lymph node metastasis is rarely presented [13], and there was no mesenteric lymphadenopathy in this patient, lymphadenectomy was not necessary. Again, as being classified as a high-risk patient, implementation of adjuvant imatinib mesylate seems to help increase the survival rate in this population [14].

## 4 Conclusion

To our knowledge, there has been a report of the heterotopic pancreas that was misdiagnosed as GISTs [15]. However, jejunal GISTs mimicking a tumor of the pancreas has not been yet reported. With an infrequent presentation of a very large tumor arose from jejunum, and mimicked a cystic tumor of the pancreas, it was difficult to initially diagnose a jejunal GIST in this case.

## Consent

The patient has been informed prior to the conduction of this manuscript and informed consent has also been obtained. A copy of the written consent is available for review by the editor-in-chief of the journal on request.

## Provenance and peer review

Not commissioned, externally peer-reviewed.

## Ethical approval

The consent form and information sheet using in the process of obtaining a consent were approved by IRB at our institution.

## Funding

This work received no funding.

## Author contribution

Kitti Wongta collected data and wrote manuscript.

Vichack Chakrapan Na Ayudhya, Kobkool Chakrapan Na Ayudhya, Rapol Poolsavatkitikool, Papot Charutragulchai, and Singha Sripreechapattana contributed to conceptualization.

Paiboon Sookpotarom and Vorapatu Tangsirapat contributed to conceptualization, data curation, supervision and editing of the manuscript.

Paisarn Vejchapipat finally edited this manuscript.

## Conflict of interest statement

None.

## Guarantor

Kitti Wongta, Vorapatu Tangsirapat, and Paiboon Sookpotarom

## Research Registration Number

NA.
